# Supplementary material for: A Likelihood Approach for Real-Time Calibration of Stochastic Compartmental Epidemic Models
Source: PLoS Comput Biol. 2017 Jan 17;13(1):e1005257. doi: 10.1371/journal.pcbi.1005257 (PMC5240920; doi:10.1371/journal.pcbi.1005257)
Supplement: S1 File — (TAR.GZ) [file pcbi.1005257.s014.tar.gz › HSPH_Online-SI-Revision/PF08/n1-extreme/n1-extreme_PF2_table0.pdf]

1	newly 3 weeks spec	79.	43.14	{8., 75.}	49.7722	0.43068	newly 3 weeks cumul	342.	151.74	{33., 245.}	55.6316	0.453662	newly 5 weeks spec	40.	29.08	{5., 58.}	48.45	0.436801	newly 5 weeks cumul	434.	216.73	{48., 377.}	50.3664	0.407834	newly 60 weeks cumul	525.	334.06	{78., 585.}	43.6229	0.342049
2	newly 3 weeks spec	61.	38.6	{17., 62.}	41.6066	0.269412	newly 3 weeks cumul	211.	129.11	{64., 202.}	41.6161	0.269041	newly 5 weeks spec	24.	29.89	{12., 55.}	51.9583	0.208201	newly 5 weeks cumul	275.	192.42	{96., 318.}	37.6291	0.228166	newly 56 weeks cumul	368.	336.53	{164., 544.}	36.1168	0.187
3	newly 3 weeks spec	93.	35.22	{6., 72.}	66.0645	0.661082	newly 3 weeks cumul	270.	117.41	{27., 225.}	59.0185	0.504873	newly 5 weeks spec	51.	26.66	{4., 55.}	57.8824	0.572845	newly 5 weeks cumul	381.	175.81	{38., 346.}	57.8924	0.498369	newly 58 weeks cumul	577.	285.37	{47., 581.}	54.74	0.487458
4	newly 3 weeks spec	61.	27.15	{2., 57.}	60.2787	0.628779	newly 3 weeks cumul	180.	93.54	{10., 182.}	55.3111	0.490087	newly 5 weeks spec	40.	19.2	{2., 45.}	59.4	0.604855	newly 5 weeks cumul	278.	135.64	{18., 271.}	56.8058	0.509454	newly 57 weeks cumul	490.	237.97	{43., 513.}	55.5286	0.503058
5	newly 3 weeks spec	56.	18.48	{5., 37.}	67.3214	0.605141	newly 3 weeks cumul	216.	67.3	{29., 120.}	68.8426	0.582686	newly 5 weeks spec	40.	13.13	{3., 25.}	67.875	0.668737	newly 5 weeks cumul	294.	96.27	{37., 186.}	67.2551	0.565546	newly 50 weeks cumul	415.	167.39	{41., 318.}	59.694	0.503487
6	newly 3 weeks spec	54.	18.57	{1., 43.}	67.0556	0.769899	newly 3 weeks cumul	153.	66.5	{6., 146.}	60.4967	0.580834	newly 5 weeks spec	31.	13.84	{0., 30.}	62.8387	0.691457	newly 5 weeks cumul	228.	96.2	{9., 214.}	61.3772	0.598919	newly 55 weeks cumul	355.	175.71	{10., 392.}	59.7775	0.579555
7	newly 3 weeks spec	68.	31.62	{8., 53.}	55.0882	0.452575	newly 3 weeks cumul	227.	110.08	{33., 176.}	52.2731	0.403043	newly 5 weeks spec	52.	23.11	{4., 42.}	60.3654	0.567558	newly 5 weeks cumul	341.	160.39	{38., 260.}	53.5748	0.427761	newly 59 weeks cumul	479.	269.31	{51., 531.}	50.9081	0.402209
8	newly 3 weeks spec	44.	25.34	{5., 42.}	47.9091	0.390338	newly 3 weeks cumul	184.	87.19	{22., 155.}	53.8207	0.416031	newly 5 weeks spec	25.	18.69	{3., 36.}	51.72	0.427902	newly 5 weeks cumul	249.	127.1	{30., 232.}	50.8675	0.394924	newly 53 weeks cumul	332.	230.63	{42., 434.}	45.503	0.340482
9	newly 3 weeks spec	65.	38.58	{8., 66.}	45.8154	0.368153	newly 3 weeks cumul	257.	132.16	{29., 234.}	49.6031	0.401434	newly 5 weeks spec	29.	27.99	{6., 54.}	51.1379	0.299692	newly 5 weeks cumul	344.	191.94	{51., 343.}	46.1977	0.362285	newly 60 weeks cumul	427.	338.57	{107., 585.}	40.6862	0.26505
10	newly 3 weeks spec	63.	26.3	{8., 46.}	58.254	0.476659	newly 3 weeks cumul	193.	89.41	{31., 152.}	54.0984	0.40911	newly 5 weeks spec	45.	18.39	{4., 36.}	59.6222	0.531906	newly 5 weeks cumul	286.	129.07	{42., 208.}	55.1503	0.423902	newly 53 weeks cumul	451.	230.49	{55., 417.}	50.6674	0.401483
11	newly 3 weeks spec	56.	34.04	{12., 59.}	45.2857	0.335506	newly 3 weeks cumul	197.	121.34	{40., 212.}	43.7563	0.303968	newly 5 weeks spec	31.	23.93	{5., 47.}	50.9355	0.374633	newly 5 weeks cumul	268.	174.15	{61., 312.}	42.9739	0.294071	newly 59 weeks cumul	345.	295.2	{86., 550.}	45.4783	0.265513
12	newly 3 weeks spec	60.	36.36	{8., 65.}	46.7333	0.40976	newly 3 weeks cumul	251.	122.57	{20., 206.}	52.1394	0.444799	newly 5 weeks spec	36.	27.79	{3., 59.}	53.8611	0.468725	newly 5 weeks cumul	329.	182.04	{32., 338.}	48.1033	0.408158	newly 60 weeks cumul	420.	301.51	{65., 589.}	44.9119	0.349421
13	newly 3 weeks spec	43.	21.49	{7., 42.}	56.814	0.469291	newly 3 weeks cumul	128.	71.93	{27., 151.}	52.4609	0.375353	newly 5 weeks spec	32.	16.01	{3., 34.}	59.1563	0.538075	newly 5 weeks cumul	184.	106.14	{37., 214.}	52.8043	0.382664	newly 53 weeks cumul	478.	198.95	{45., 391.}	59.9895	0.524689
14	newly 3 weeks spec	35.	40.49	{17., 74.}	47.2286	0.20588	newly 3 weeks cumul	175.	135.78	{57., 231.}	38.6514	0.229657	newly 5 weeks spec	20.	29.69	{12., 55.}	69.45	0.246981	newly 5 weeks cumul	217.	200.34	{88., 356.}	38.5899	0.198753	newly 57 weeks cumul	250.	343.34	{137., 587.}	59.144	0.220383
15	newly 3 weeks spec	59.	22.37	{7., 35.}	62.8305	0.536967	newly 3 weeks cumul	186.	76.78	{33., 134.}	59.5699	0.460978	newly 5 weeks spec	32.	15.87	{3., 30.}	54.2813	0.501921	newly 5 weeks cumul	268.	111.79	{44., 196.}	59.0261	0.463769	newly 52 weeks cumul	342.	209.7	{47., 423.}	47.0877	0.35992
16	newly 3 weeks spec	56.	33.67	{9., 59.}	47.375	0.397593	newly 3 weeks cumul	175.	112.72	{25., 190.}	43.0743	0.340794	newly 5 weeks spec	29.	25.12	{4., 51.}	55.931	0.397675	newly 5 weeks cumul	242.	167.67	{34., 311.}	43.5	0.338987	newly 59 weeks cumul	393.	286.	{57., 489.}	43.2061	0.334146
17	newly 3 weeks spec	48.	33.42	{13., 60.}	43.75	0.2786	newly 3 weeks cumul	177.	109.73	{42., 179.}	45.4068	0.298494	newly 5 weeks spec	42.	25.4	{9., 49.}	48.1905	0.333994	newly 5 weeks cumul	264.	164.63	{60., 302.}	44.7538	0.294959	newly 56 weeks cumul	488.	308.96	{102., 546.}	43.3689	0.29889
18	newly 3 weeks spec	77.	34.51	{6., 60.}	56.7143	0.50541	newly 3 weeks cumul	277.	118.46	{23., 200.}	57.3357	0.49557	newly 5 weeks spec	51.	25.72	{2., 51.}	58.0784	0.569257	newly 5 weeks cumul	383.	174.38	{38., 311.}	55.0601	0.479811	newly 59 weeks cumul	532.	297.	{57., 612.}	50.188	0.433267
19	newly 3 weeks spec	85.	36.43	{8., 61.}	57.2588	0.496501	newly 3 weeks cumul	252.	124.65	{30., 203.}	51.2976	0.410946	newly 5 weeks spec	73.	27.38	{3., 58.}	63.4795	0.670627	newly 5 weeks cumul	405.	183.45	{45., 300.}	54.8914	0.453494	newly 60 weeks cumul	563.	308.64	{73., 572.}	49.4103	0.402282
20	newly 3 weeks spec	46.	24.64	{6., 42.}	51.087	0.411147	newly 3 weeks cumul	195.	88.84	{31., 154.}	55.2821	0.421548	newly 5 weeks spec	24.	17.81	{3., 33.}	51.625	0.382343	newly 5 weeks cumul	257.	127.43	{41., 229.}	52.3774	0.397941	newly 53 weeks cumul	327.	212.82	{52., 367.}	47.0887	0.346286
21	newly 3 weeks spec	59.	34.81	{12., 67.}	48.5254	0.357108	newly 3 weeks cumul	155.	115.65	{40., 214.}	45.8387	0.275113	newly 5 weeks spec	46.	25.66	{6., 54.}	53.1739	0.477776	newly 5 weeks cumul	257.	171.02	{61., 320.}	46.4125	0.308055	newly 56 weeks cumul	478.	306.84	{83., 593.}	45.8787	0.334756
22	newly 3 weeks spec	49.	26.7	{4., 53.}	52.6122	0.470847	newly 3 weeks cumul	172.	96.57	{19., 190.}	50.3198	0.413019	newly 5 weeks spec	32.	17.83	{2., 38.}	53.4063	0.489057	newly 5 weeks cumul	242.	136.78	{26., 279.}	50.686	0.413018	newly 57 weeks cumul	358.	222.52	{39., 442.}	49.3743	0.391559
23	newly 3 weeks spec	51.	33.59	{5., 73.}	51.9412	0.439402	newly 3 weeks cumul	194.	111.14	{17., 228.}	49.1031	0.435701	newly 5 weeks spec	37.	25.73	{1., 63.}	57.5946	0.567136	newly 5 weeks cumul	294.	166.46	{26., 352.}	50.3673	0.456843	newly 59 weeks cumul	412.	294.91	{37., 613.}	50.4248	0.420945
24	newly 3 weeks spec	65.	39.77	{8., 65.}	43.8615	0.416334	newly 3 weeks cumul	306.	141.94	{27., 234.}	53.6144	0.438765	newly 5 weeks spec	35.	27.16	{0., 53.}	50.1143	0.486217	newly 5 weeks cumul	386.	201.56	{35., 335.}	48.3212	0.400215	newly 60 weeks cumul	448.	316.94	{50., 537.}	40.8884	0.318885
25	newly 3 weeks spec	55.	26.7	{3., 57.}	56.7636	0.567855	newly 3 weeks cumul	189.	90.13	{13., 185.}	55.6561	0.51093	newly 5 weeks spec	45.	19.66	{2., 44.}	60.6667	0.642424	newly 5 weeks cumul	280.	132.13	{18., 274.}	55.9036	0.515031	newly 57 weeks cumul	488.	232.61	{32., 484.}	56.0184	0.526617
26	newly 3 weeks spec	68.	40.64	{11., 69.}	44.1176	0.310782	newly 3 weeks cumul	336.	144.64	{51., 244.}	56.9524	0.429847	newly 5 weeks spec	22.	26.63	{8., 48.}	57.1364	0.265335	newly 5 weeks cumul	403.	206.24	{67., 343.}	48.8536	0.355331	newly 59 weeks cumul	451.	308.9	{120., 476.}	36.9933	0.241154
27	newly 3 weeks spec	39.	26.87	{8., 49.}	45.4103	0.319979	newly 3 weeks cumul	136.	91.75	{38., 165.}	43.9632	0.282078	newly 5 weeks spec	42.	21.81	{4., 42.}	53.2619	0.464733	newly 5 weeks cumul	218.	137.66	{52., 242.}	44.6514	0.303989	newly 54 weeks cumul	436.	255.61	{59., 445.}	48.3417	0.36622
28	newly 3 weeks spec	51.	28.39	{7., 49.}	50.6078	0.414554	newly 3 weeks cumul	228.	101.54	{28., 166.}	55.7807	0.448113	newly 5 weeks spec	34.	20.43	{1., 34.}	54.2059	0.531936	newly 5 weeks cumul	314.	146.41	{36., 258.}	55.2197	0.449126	newly 58 weeks cumul	442.	232.7	{49., 405.}	53.2624	0.427522
29	newly 3 weeks spec	80.	34.15	{11., 58.}	57.5125	0.465685	newly 3 weeks cumul	289.	121.2	{46., 213.}	58.0623	0.444375	newly 5 weeks spec	36.	24.28	{6., 45.}	44.8333	0.374383	newly 5 weeks cumul	356.	174.14	{61., 304.}	51.2022	0.378999	newly 55 weeks cumul	420.	298.45	{69., 499.}	39.2452	0.2717
30	newly 3 weeks spec	25.	14.34	{1., 27.}	52.	0.532472	newly 3 weeks cumul	94.	51.06	{8., 95.}	51.5319	0.454814	newly 5 weeks spec	18.	10.71	{0., 23.}	58.5	0.613686	newly 5 weeks cumul	134.	73.69	{10., 136.}	51.2463	0.465505	newly 50 weeks cumul	194.	131.19	{12., 272.}	55.9124	0.496525
31	newly 3 weeks spec	69.	33.71	{11., 60.}	52.913	0.417115	newly 3 weeks cumul	249.	120.33	{46., 211.}	52.2129	0.397653	newly 5 weeks spec	42.	23.14	{5., 42.}	53.4762	0.446042	newly 5 weeks cumul	339.	172.67	{63., 312.}	50.469	0.379136	newly 59 weeks cumul	419.	287.1	{90., 502.}	43.0024	0.295234
32	newly 3 weeks spec	64.	33.05	{8., 59.}	50.7031	0.427671	newly 3 weeks cumul	288.	119.89	{29., 205.}	58.3715	0.474834	newly 5 weeks spec	34.	22.06	{3., 42.}	48.5882	0.440255	newly 5 weeks cumul	366.	169.29	{50., 311.}	53.8115	0.428157	newly 59 weeks cumul	414.	277.95	{71., 491.}	43.1184	0.319646
33	newly 3 weeks spec	77.	29.58	{12., 51.}	61.7922	0.487075	newly 3 weeks cumul	224.	101.95	{42., 174.}	55.4688	0.404005	newly 5 weeks spec	47.	21.58	{5., 40.}	56.0426	0.468382	newly 5 weeks cumul	333.	148.22	{62., 255.}	56.2282	0.413543	newly 55 weeks cumul	564.	268.62	{84., 458.}	53.4752	0.405046
34	newly 3 weeks spec	60.	30.95	{4., 64.}	53.85	0.519337	newly 3 weeks cumul	219.	106.55	{17., 217.}	52.7443	0.484018	newly 5 weeks spec	38.	22.02	{2., 50.}	54.4211	0.554469	newly 5 weeks cumul	306.	155.4	{22., 311.}	52.1699	0.480696	newly 58 weeks cumul	433.	266.65	{28., 536.}	48.7875	0.438695
35	newly 3 weeks spec	68.	31.53	{5., 54.}	55.5441	0.54188	newly 3 weeks cumul	222.	107.78	{21., 187.}	52.4865	0.493616	newly 5 weeks spec	42.	24.32	{2., 55.}	56.1905	0.577145	newly 5 weeks cumul	317.	160.47	{29., 311.}	51.8076	0.498618	newly 59 weeks cumul	467.	268.65	{43., 553.}	50.5803	0.478299
36	newly 3 weeks spec	69.	27.74	{2., 65.}	62.9275	0.7163	newly 3 weeks cumul	224.	98.52	{6., 202.}	60.6696	0.633741	newly 5 weeks spec	46.	19.16	{1., 44.}	63.4348	0.728935	newly 5 weeks cumul	333.	141.28	{10., 312.}	61.0571	0.651392	newly 56 weeks cumul	462.	235.16	{16., 508.}	55.1861	0.588252
37	newly 3 weeks spec	58.	20.73	{5., 41.}	64.3621	0.598538	newly 3 weeks cumul	192.	72.84	{24., 136.}	62.0625	0.506034	newly 5 weeks spec	31.	14.69	{3., 32.}	57.9032	0.522643	newly 5 weeks cumul	273.	105.07	{36., 205.}	61.5128	0.507764	newly 51 weeks cumul	413.	184.34	{44., 364.}	56.2906	0.476356
38	newly 3 weeks spec	60.	37.46	{17., 66.}	44.4667	0.295446	newly 3 weeks cumul	244.	122.38	{51., 212.}	52.2131	0.371866	newly 5 weeks spec	40.	30.18	{9., 53.}	41.9	0.288564	newly 5 weeks cumul	319.	186.06	{82., 315.}	45.4671	0.307478	newly 56 weeks cumul	406.	360.93	{135., 587.}	37.4951	0.211689
39	newly 3 weeks spec	93.	38.08	{3., 75.}	59.1398	0.591083	newly 3 weeks cumul	314.	124.75	{13., 235.}	60.2707	0.577857	newly 5 weeks spec	46.	28.73	{1., 61.}	50.9783	0.534712	newly 5 weeks cumul	440.	185.96	{18., 361.}	57.7364	0.553023	newly 59 weeks cumul	558.	323.49	{20., 602.}	46.3459	0.441523
40	newly 3 weeks spec	44.	25.48	{6., 53.}	52.3182	0.424292	newly 3 weeks cumul	165.	91.89	{20., 188.}	50.7697	0.394417	newly 5 weeks spec	31.	18.26	{2., 40.}	55.0968	0.513168	newly 5 weeks cumul	230.	131.82	{24., 266.}	51.0435	0.396683	newly 57 weeks cumul	342.	216.65	{43., 473.}	51.7047	0.400656
41	newly 3 weeks spec	54.	20.18	{3., 38.}	62.7037	0.634371	newly 3 weeks cumul	164.	65.84	{16., 122.}	59.8537	0.517202	newly 5 weeks spec	33.	14.38	{0., 31.}	60.	0.701899	newly 5 weeks cumul	248.	96.35	{17., 182.}	61.1895	0.554448	newly 52 weeks cumul	369.	187.77	{17., 376.}	53.477	0.520819
42	newly 3 weeks spec	75.	41.27	{10., 67.}	49.4533	0.396973	newly 3 weeks cumul	284.	145.96	{41., 239.}	49.	0.369254	newly 5 weeks spec	55.	28.58	{4., 56.}	52.2545	0.500235	newly 5 weeks cumul	401.	208.06	{62., 360.}	48.8429	0.371294	newly 60 weeks cumul	505.	316.07	{74., 577.}	43.8	0.323045
43	newly 3 weeks spec	82.	37.	{11., 60.}	55.4146	0.466693	newly 3 weeks cumul	252.	123.43	{34., 200.}	51.5278	0.406057	newly 5 weeks spec	62.	26.82	{5., 51.}	59.2903	0.54418	newly 5 weeks cumul	378.	182.43	{50., 311.}	52.209	0.423536	newly 59 weeks cumul	545.	305.78	{68., 602.}	48.3486	0.39343
44	newly 3 weeks spec	70.	29.44	{4., 56.}	59.1143	0.536849	newly 3 weeks cumul	222.	104.92	{23., 189.}	53.6306	0.441446	newly 5 weeks spec	43.	19.83	{4., 43.}	58.1163	0.540445	newly 5 weeks cumul	326.	149.46	{35., 279.}	55.	0.458976	newly 57 weeks cumul	430.	232.02	{45., 442.}	51.5721	0.421871
45	newly 3 weeks spec	80.	38.82	{8., 79.}	53.325	0.436467	newly 3 weeks cumul	274.	133.02	{34., 235.}	52.2336	0.414961	newly 5 weeks spec	45.	27.67	{5., 54.}	49.5333	0.424611	newly 5 weeks cumul	386.	192.92	{54., 357.}	51.0674	0.402378	newly 60 weeks cumul	569.	327.52	{104., 585.}	46.6292	0.356791
46	newly 3 weeks spec	54.	33.37	{8., 59.}	51.0926	0.379996	newly 3 weeks cumul	225.	111.4	{29., 189.}	54.1689	0.416091	newly 5 weeks spec	46.	23.08	{4., 47.}	57.	0.511102	newly 5 weeks cumul	335.	162.32	{41., 304.}	55.0925	0.43207	newly 57 weeks cumul	485.	262.87	{67., 527.}	51.2103	0.398458
47	newly 3 weeks spec	69.	33.29	{1., 72.}	61.	0.627327	newly 3 weeks cumul	202.	110.41	{19., 225.}	53.4802	0.458465	newly 5 weeks spec	59.	24.2	{0., 52.}	64.4746	0.747506	newly 5 weeks cumul	320.	164.19	{20., 331.}	56.0531	0.502132	newly 58 weeks cumul	544.	268.83	{28., 573.}	55.4063	0.529027
48	newly 3 weeks spec	53.	29.77	{1., 63.}	62.1321	0.629069	newly 3 weeks cumul	180.	97.54	{8., 205.}	57.2889	0.524151	newly 5 weeks spec	40.	21.99	{0., 50.}	62.275	0.688747	newly 5 weeks cumul	270.	145.98	{9., 312.}	58.8593	0.547285	newly 58 weeks cumul	460.	253.17	{11., 518.}	55.1804	0.537858
49	newly 3 weeks spec	56.	37.36	{8., 64.}	42.7857	0.321347	newly 3 weeks cumul	258.	137.75	{40., 234.}	47.7791	0.359695	newly 5 weeks spec	43.	25.49	{5., 49.}	51.	0.465632	newly 5 weeks cumul	341.	193.76	{54., 332.}	45.5249	0.339899	newly 60 weeks cumul	475.	294.76	{73., 505.}	43.6295	0.322886
50	newly 3 weeks spec	49.	27.12	{2., 60.}	58.9388	0.55859	newly 3 weeks cumul	150.	91.4	{12., 194.}	53.24	0.446475	newly 5 weeks spec	26.	18.59	{1., 42.}	60.3462	0.559837	newly 5 weeks cumul	211.	132.63	{18., 265.}	53.7488	0.450279	newly 58 weeks cumul	361.	240.04	{20., 505.}	53.6787	0.457939
